# Association of peripheral differential leukocyte counts with dyslipidemia risk in Chinese patients with hypertension: insight from the China Stroke Primary Prevention Trial[S]

**DOI:** 10.1194/jlr.P067686

**Published:** 2016-12-29

**Authors:** Yanhong Liu, Xiangyi Kong, Wen Wang, Fangfang Fan, Yan Zhang, Min Zhao, Yi Wang, Yupeng Wang, Yu Wang, Xianhui Qin, Genfu Tang, Binyan Wang, Xiping Xu, Fan Fan Hou, Wei Gao, Ningling Sun, Jianping Li, Scott A. Venners, Shanqun Jiang, Yong Huo

**Affiliations:** School of Life Sciences,* Anhui University, Hefei, China; Department of Cardiology,† Peking University First Hospital, Beijing, China; Institute for Biomedicine,‡ Anhui Medical University, Hefei, China; National Clinical Research Study Center for Kidney Disease,§ State Key Laboratory for Organ Failure Research, Renal Division, Nanfang Hospital, Southern Medical University, Guangzhou, China; Department of Neurology,‖ Huashan Hospital, Fudan University, Shanghai, China; Department of Cardiology,# Peking University Third Hospital, Beijing, China; Department of Cardiology,** Peking University People’s Hospital, Beijing, China; Faculty of Health Sciences,†† Simon Fraser University, Burnaby, BC, Canada

**Keywords:** total leukocyte, neutrophil, lymphocyte, epidemiology, CSPPT

## Abstract

The aim of the present study was to examine the association between peripheral differential leukocyte counts and dyslipidemia in a Chinese hypertensive population. A total of 10,866 patients with hypertension were enrolled for a comprehensive assessment of cardiovascular risk factors using data from the China Stroke Primary Prevention Trial. Plasma lipid levels and total leukocyte, neutrophil, and lymphocyte counts were determined according to standard methods. Peripheral differential leukocyte counts were consistently and positively associated with serum total cholesterol (TC), LDL cholesterol (LDL-C), and TG levels (all *P* < 0.001 for trend), while inversely associated with HDL cholesterol levels (*P* < 0.05 for trend). In subsequent analyses where serum lipids were dichotomized (dyslipidemia/normolipidemia), we found that patients in the highest quartile of total leukocyte count (≥7.6 × 10^9^ cells/l) had 1.64 times the risk of high TG [95% confidence interval (CI): 1.46, 1.85], 1.34 times the risk of high TC (95% CI: 1.20, 1.50), and 1.24 times the risk of high LDL-C (95% CI: 1.12, 1.39) compared with their counterparts in the lowest quartile of total leukocyte count. Similar patterns were also observed with neutrophils and lymphocytes. In summary, these findings indicate that elevated differential leukocyte counts are directly associated with serum lipid levels and increased odds of dyslipidemia.

Atherosclerosis is a multi-step chronic inflammatory disease. Largely characterized by the formation of lipid and immune-cell-containing plaques in the intima of large and mid-sized arteries (1), it is often the underlying cause of CVDs and stroke (1–3).

Traditionally, atherosclerosis was thought to result from passive lipid accumulation in the arterial vessel wall. However, a growing body of evidence suggests that the pathogenesis of atherosclerosis is much more complex than formerly believed. Immune cells and inflammation play a key role in its pathogenesis in conjunction with hyperlipidemia (4). However, how lipids interact with the immune system is largely unknown.

There is some limited evidence (5, 6) showing that increased levels of LDL promote cholesterol accumulation in the matrix beneath the endothelial cell layer of blood vessels (7) and promote an inflammatory response in the artery wall, driving the process of atherosclerosis. This is opposed by HDL, which promotes cellular efflux of cholesterol and reduces inflammation (5). The early inflammatory response to retained cholesterol is enhanced by oxidative modification of the LDL cholesterol (LDL-C) trapped in the vessel wall (7). Accumulated oxidized LDL leads to decreased cholesterol efflux from macrophage foam cells (8), amplifies the toll-like receptor signaling in macrophages (9, 10), and activates endothelial cells. Activation of arterial endothelial cells leads to recruitment of blood-borne monocytes into atherosclerotic lesion sites within the vessel wall (11, 12) and augments the production of cytokines and chemokines (7) that interact with cognate chemokine receptors on monocytes. Accumulation of monocytes and monocyte-derived ma­crophages in the wall of large arteries leads to chronic inflammation and the development and progression of atherosclerosis.

A large number of epidemiological studies has intensively evaluated multiple markers of inflammation as potential risk factors for the development of atherosclerosis and CVDs, such as high-sensitivity C-reactive protein (hsCRP), interleukin-6, and total leukocyte and its subset counts [neutrophil counts (13, 14), monocyte counts (15), and lymphocyte percentages (15)]. Previous studies have found strong evidence of association between the frequency of leukocytes and/or its subsets, or the neutrophil/lymphocyte ratio (NLR) and vascular disease morbidity and mortality. Misialek et al. (16) prospectively examined the relationship between total white blood cell (WBC) count with incident atrial fibrillation (AF) in the Atherosclerosis Risk in Communities study and found that high total WBC, neutrophil, and monocyte counts were each associated with higher AF risk, while lymphocyte count was inversely associated with AF risk. A study by Sharma et al. (17) indicated that among patients with evidence of acute coronary syndrome, those who were hypertensive, diabetic, or habitual smokers had significantly higher levels of total WBC, neutrophil, NLR, and platelet/lymphocyte ratio. The authors also demonstrated that neutrophil, lymphocyte, and total WBC counts, along with their ratios, predicted mortality.

However, there is a dearth of reports on the association of total leukocyte and its subset counts with cardiovascular risk factors leading to atherosclerotic vascular diseases in Asian populations. Recently, a cross-sectional study from a population of 2,953 healthy Japanese men and women (18) showed that lymphocyte count was significantly associated with high LDL-C, high TG, and low HDL cholesterol (HDL-C) in men and high LDL-C in women. Moreover, a prospective investigation from a Chinese population involving 1,287 patients with a mean age of 58 years provided convincing evidence that both neutrophil count and the Global Registry of Acute Coronary Events risk score are independent and joint predictors for major adverse cardiovascular events in patients with ST-elevation myocardial infarction (19).

Our present study design is the first large-scale cross-sectional study in a Chinese hypertensive population to investigate the association between peripheral differential leukocyte counts (total leukocytes, neutrophils, and lymphocytes) and lipid profiles. We hypothesized that participants with elevated leukocyte counts would have increased dyslipidemia.

## RESEARCH DESIGN AND METHODS

### Study population

All subjects came from the China Stroke Primary Prevention Trial (CSPPT, clinicaltrials.gov identifier: NCT00794885). The CSPPT was a large community-based, randomized, double-blind, and parallel-controlled trial with a total of 20,702 participants. It was designed to evaluate whether combination therapy with enalapril and folic acid is more effective in reducing first stroke than enalapril alone among Chinese adults with hypertension. Participants in the CSPPT study were deemed “relatively healthy” hypertensives without histories of myocardial infarction, stroke, heart failure, cancer, or serious mental disorder. Details regarding the inclusion/exclusion criteria, treatment assignment, and outcome measures of the trial have been previously described (http://clinicaltrials.gov/ct2/show/NCT00794885).

**Fig. 1.**
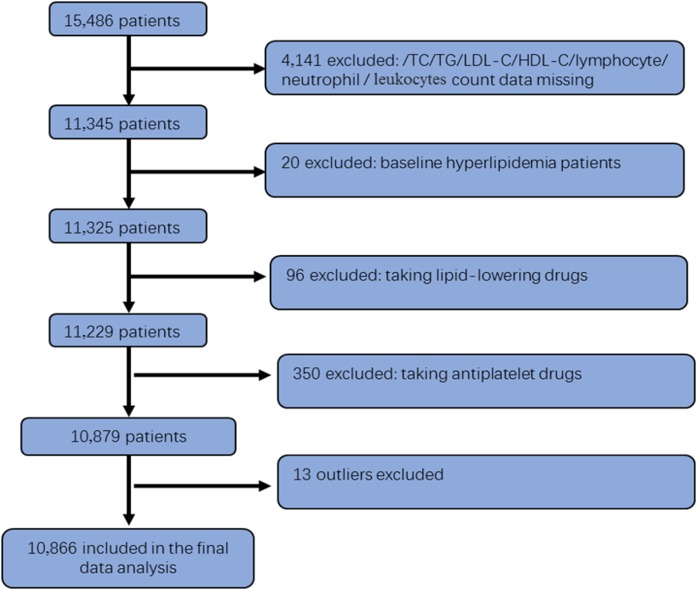
The flow chart of population selection. In total, 15,486 patients were recruited from the Lianyungang region of Jiangsu Province. Among them, 11,345 participants had complete data on lipid measurements and total leukocyte, neutrophil, and lymphocyte counts, after excluding 446 participants who were taking anti-platelet and anti-hyperlipidemic medication. After excluding 13 outliers of leukocyte, neutrophil, and lymphocyte counts, the final analytic sample of 10,866 participants was obtained.

In the present study, 15,486 patients were recruited from the Lianyungang region of Jiangsu Province. Among them, 11,345 participants had complete data on lipid measurements and total leukocyte, neutrophil, and lymphocyte counts, after excluding 446 participants who were taking anti-platelet and anti-hyperlipidemic medication. Moreover, after 13 outliers of leukocyte, neutrophil, and lymphocyte counts were excluded, the final analytic sample of 10,866 participants was obtained (as shown in **Fig. 1**). The present study was approved by the Ethics Committee of the Institute of Biomedicine, Anhui Medical University, Hefei, China. Written informed consent was obtained from each participant before data collection.

### Questionnaire

All participants were administered a standardized questionnaire that requested information on occupation, medical history, past and current medications, and personal habits such as cigarette smoking and alcohol consumption.

### Measurements

After an overnight fast, a venous blood sample was obtained from each subject. Serum or plasma samples were separated within 30 min of collection and stored at −70°C. The serum levels of TG, total cholesterol (TC), LDL-C, HDL-C, and fasting glucose were determined enzymatically with a commercially available assay kit (Hitachi P800, Holliston, MA). A complete blood count analysis including leukocytes was performed within 2 h of collection using a Beckman Coulter Gen-S automated analyzer (High Wycombe, UK), following the hospital laboratory policy. Resting seated blood pressure (BP) measurements were obtained using a mercury sphygmomanometer with an appropriate cuff size. BP was measured three times, with a 5 min rest period in between each and the average of the three measurements was used for statistical analyses.

### Statistical analysis

All analyses were performed using EmpowerStats (http://www.empowerstats.com) and the statistical package R (3.2.3 version). Leukocyte counts were divided into quartiles to create a categorical variable. Data were presented as mean ± SD or proportions. Comparisons between groups were performed using chi-square tests for categorical variables and ANOVA for continuous variables. Because baseline leukocyte counts had a skewed distribution, multiple linear regression analyses were used to assess the associations between log-transformed baseline leukocyte counts and serum lipid levels (including log-transformed TC and HDL-C). Odds ratios (ORs) and 95% confidence intervals (CIs) of those having high lipid levels were estimated by multiple logistic regression analyses with the lowest quartile as the reference class. Adjusted smoothing spline plots of lipid levels by leukocyte counts were created. Multiple logistic regression analyses were also used to verify an interaction scale for diabetes × leukocyte counts categories on lipid profiles. Diabetes was defined as a fasting plasma glucose concentration greater than or equal to 7.0 mmol/l (20), or self-reported diabetes paired with the use of hypoglycemic medication. Serum lipids were dichotomized (dyslipidemia/normolipidemia). High TC was defined as TC ≥200 mg/dl; high TG was defined as TG ≥150 mg/dl; high LDL-C was defined as LDL-C ≥130 mg/dl; and low HDL-C was defined as HDL-C ≤40 mg/dl (21). Patients with diastolic BP (DBP) ≥90 mmHg or systolic BP (SBP) ≥140 mmHg or, who were currently taking antihypertensive medication, were defined as having hypertension (22). Trend tests were calculated by modeling the differential leukocyte count quartile categories as continuous variables. A two-sided *P* value <0.05 was considered to be significant.

## RESULTS

### Baseline characteristics

The cross-sectional population in the current study consisted of 10,866 hypertensive patients with an average age of 59.5 ± 7.6 years (**Table 1**). The mean total leukocyte count was 6.6 ± 1.8 × 10^9^ cells/l (median = 6.3 × 10^9^ cells/l; ranged from 0.6 × 10^9^ cells/l to 17.1 × 10^9^ cells/l); the mean neutrophil count was 3.9 ± 1.4 × 10^9^ cells/l (median = 3.7 × 10^9^ cells/l; ranged from 0.3 × 10^9^ cells/l to 13.1 × 10^9^ cells/l); the mean lymphocyte count was 2.1 ± 0.6 × 10^9^ cells/l (median = 2.0 × 10^9^ cells/l; ranged from 0.3 × 10^9^ cells/l to 6.3 × 10^9^ cells/l). The baseline demographic and clinical characteristics and laboratory measurements of the enrolled subjects by quartile of total leukocyte, neutrophil, and lymphocyte counts are summarized in supplemental Tables S1–S3. In summary, patients with higher total leukocyte, neutrophil, and lymphocyte counts consistently showed higher LDL-C, TC, and TG levels, but lower HDL-C levels.

**TABLE 1.** Baseline demographic and clinical parameters in total hypertensive patients

Variables	Mean ± SD
Anthropometrics	
N	10,866
Age (years)	59.5 ± 7.6
SBP (mmHg)	168.1 ± 20.8
DBP (mmHg)	95.0 ± 11.8
BMI (kg/m^2^)	25.6 ± 3.6
Platelet (10^9^/l)	256.6 ± 90.9
RBC (10^12^/l)	4.7 ± 0.7
Lymphocyte (10^9^/l)	2.1 ± 0.6
Neutrophil (10^9^/l)	3.9 ± 1.4
Total leukocytes (10^9^/l)	6.6 ± 1.8
CREA (μmol/l)	65.1 ± 18.8
GLU (mmol/l)	6.1 ± 1.8
Albumin (g/l)	49.2 ± 5.6
TC (mg/dl)	218.6 ± 45.2
TG (mg/dl)	143.6 ± 66.1
LDL-C (mg/dl)	138.8 ± 40.9
HDL-C (mg/dl)	51.3 ± 14.1
Diabetes [N (%)]	1,474 (13.6)
Sex [N (%)]	
Male	4,157 (38.3)
Female	6,709 (61.7)
Smoking status [N (%)]	
Never	7,688 (70.8)
Former	824 (7.6)
Current	2,352 (21.7)
Alcohol consumption [N (%)]	
Never	7,800 (71.8)
Former	715 (6.6)
Current	2,348 (21.6)
Drug treatment [N, (%)]	
Antihypertensive drug use	5,203 (47.9)
Beta-blocker	103 (0.9)
Diuretics	252 (2.3)
Angiotensin receptor blocker	12 (0.1)
Calcium channel blockers	693 (6.4)
ACE-inhibitors	898 (8.3)
Glucose-lowering drugs	177 (1.6)

GLU, glucosamine; ACE, angiotensin converting enzyme; CREA, creatinine; RBC, red blood cell.

### Association between differential leukocyte counts and baseline lipid parameters

**Fig. 2.**
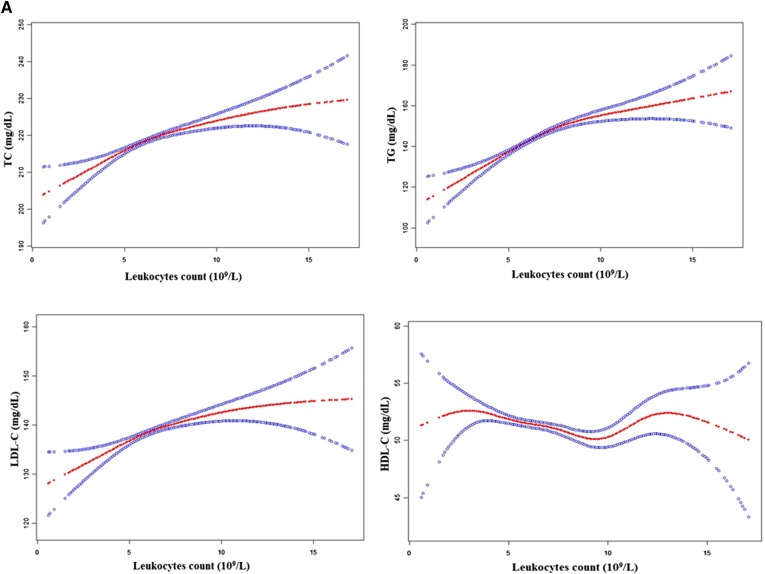

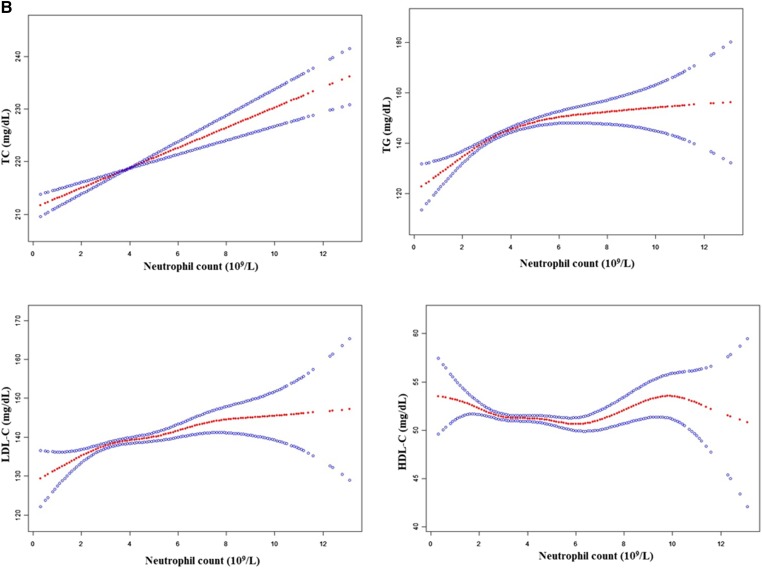

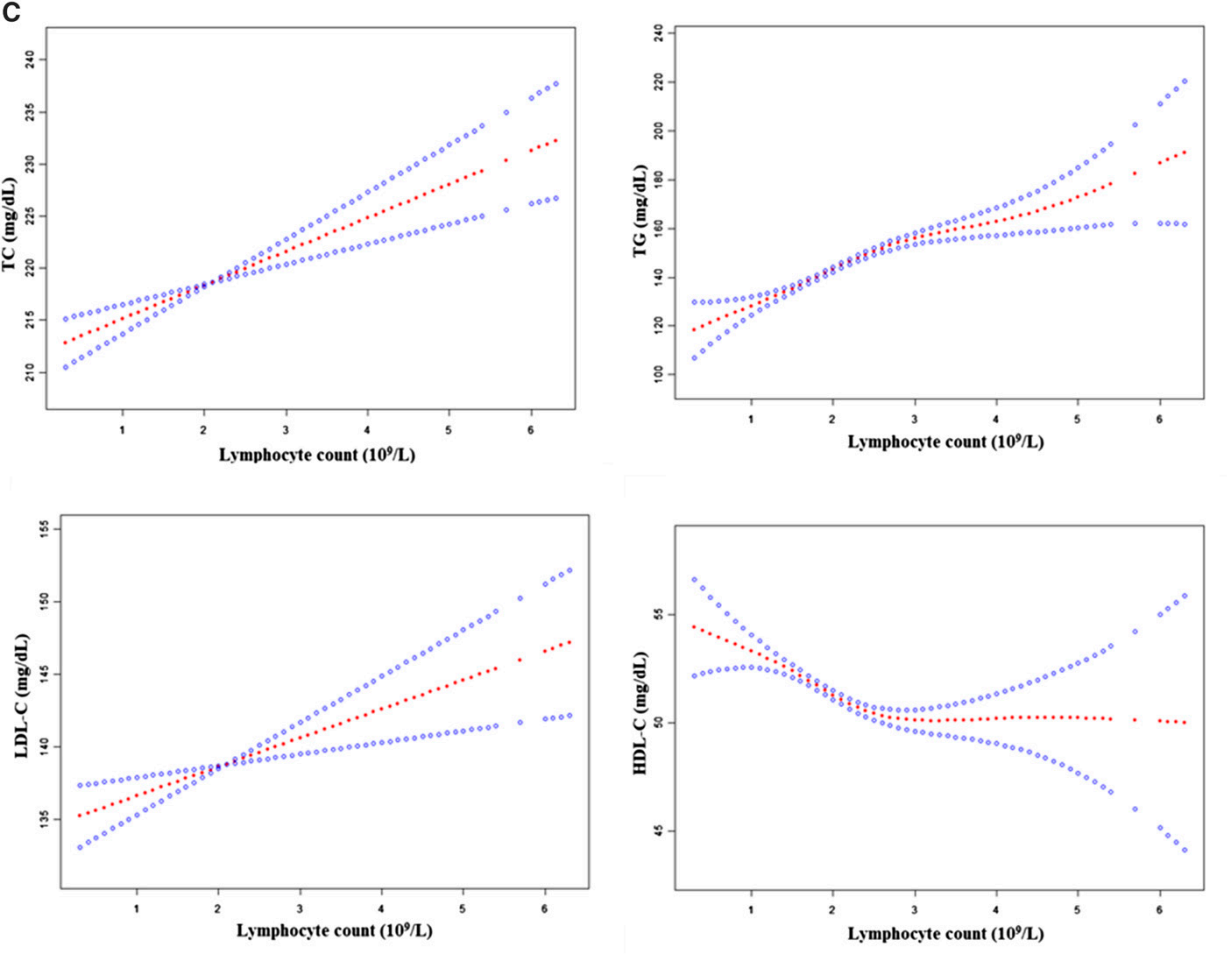
A: Multivariate adjusted smoothing spline plots of baseline lipid profiles by leukocyte count. Red dotted lines represent the spline plots of leukocyte counts and blue dotted lines represent the 95% CIs of the spline plots. Adjusted for sex, age, smoking status, alcohol consumption, SBP, DBP, BMI, and diabetes. B: Multivariate adjusted smoothing spline plots of baseline lipid profiles by neutrophil count. Red dotted lines represent the spline plots of neutrophil counts and blue dotted lines represent the 95% CIs of the spline plots. Adjusted for sex, age, smoking status, alcohol consumption, SBP, DBP, BMI, and diabetes. C: Multivariate adjusted smoothing spline plots of baseline lipid profiles by lymphocyte count. Red dotted lines represent the spline plots of lymphocyte counts and blue dotted lines represent the 95% CIs of the spline plots. Adjusted for sex, age, smoking status, alcohol consumption, SBP, DBP, BMI, and diabetes.

When analyzed as continuous variables, multiple linear regression models showed that baseline TC, TG, and LDL-C levels were positively associated with total leukocyte, neutrophil, and lymphocyte counts after adjusting for sex, age, baseline SBP, baseline DBP, BMI, alcohol consumption, smoking status, and diabetic status (all *P* < 0.05). However, baseline HDL-C level was inversely associated with total leukocyte, neutrophil, and lymphocyte counts after adjustment for the confounding factors listed above (supplemental Tables S4–S6). We found a dose-response association between baseline TC, TG, LDL-C, and HDL-C levels, and differential leukocyte counts. That is, the *P* for trend tests was statistically significant for baseline TC, TG, LDL-C, and HDL-C levels without a clear threshold. Multivariate adjusted smoothing spline plots suggest that serum TC, TG, and LDL-C levels increased with increasing total leukocyte, neutrophil, and lymphocyte counts, while HDL-C levels decreased as total leukocyte, neutrophil, and lymphocyte counts increased (as shown in **Fig. 2A–C**). Similar to differential leukocyte counts, baseline TC, TG, and LDL-C levels were positively associated with erythrocyte or platelet counts after adjusting for multiple covariables, while baseline HDL-C levels were inversely associated with erythrocyte counts (supplemental Tables S7, S8). We also detected an association between NLR and lipid profiles, but almost no significant trends were observed (supplemental Table S9).

### Association between differential leukocyte counts and dyslipidemia odds

Each serum lipid variable was then analyzed as a binary variable (low/high) using multivariate logistic regression. As shown in **Tables 2–4**, multivariate logistic regression analyses demonstrated that, after adjustment for age and sex and using the lowest quartile of total leukocytes as the reference, the ORs of having high TC increased in parallel with the quartiles of total leukocytes (ORs were 1.17, 1.39, and 1.41 from the second to the fourth quartiles, respectively, *P* < 0.001 for trend). The ORs of having high TG were 1.37, 1.67, and 1.85 from the second to the fourth quartiles, respectively (*P* < 0.001 for trend). The ORs of having high LDL-C were 1.18, 1.27, and 1.31 from the second to the fourth quartiles, respectively (*P* < 0.001 for trend). The ORs of having low HDL-C were 1.11, 1.18 and 1.23 from the second to the fourth quartiles, respectively (*P* < 0.001 for trend). Similar patterns between leukocyte subtypes (neutrophils and lymphocytes) and high TC, TG, and LDL-C levels remained significant after adjustment for confounding factors, including sex, age, baseline SBP, baseline DBP, BMI, alcohol consumption, smoking status, diabetic status, and previous medications (all adjusted *P* values <0.05), although the results did not hold for low HDL-C levels.

**TABLE 2.** Adjusted ORs (95% CI) for the association between quartiles of total leukocyte count and dyslipidemia by multivariate logistic regression models

Leukocyte Count (×10^9^ cells/l)	Serum Lipids [Low/High (N)]	Model I*^a^*[OR (95% CI) *P*]	*P* for Trend	Model II*^b^*[OR (95% CI) *P*	*P* for Trend
TC status*^c^*					
Q1 (0.6–5.3)	1,046/1,541	1	<0.001	1	<0.001
Q2 (5.4–6.3)	970/1,660	1.17 (1.05, 1.31) 0.005		1.15 (1.03, 1.29) 0.016	
Q3 (6.4–7.5)	926/1,874	1.39 (1.24, 1.55) <0.001		1.32(1.18,1.48) <0.001	
Q4 (7.6–17.1)	930/1,919	1.41 (1.26, 1.57) <0.001		1.34(1.20,1.50) <0.001	
TG status*^d^*					
Q1 (0.6–5.3)	1,819/768	1	<0.001	1	<0.001
Q2 (5.4–6.3)	1,682/948	1.37 (1.22, 1.54) <0.001		1.27 (1.13, 1.44) <0.001	
Q3 (6.4–7.5)	1,667/1,133	1.67 (1.49, 1.87) <0.001		1.48(1.31, 1.66) <0.001	
Q4 (7.6–17.1)	1,623/1,226	1.85 (1.65, 2.07) <0.001		1.64(1.46, 1.85) <0.001	
LDL-C status*^e^*					
Q1 (0.6–5.3)	1,243/1,344	1	<0.001	1	<0.001
Q2 (5.4–6.3)	1,161/1,469	1.18 (1.06, 1.31) 0.004		1.15 (1.03, 1.28) 0.014	
Q3 (6.4–7.5)	1,178/1,622	1.27 (1.14, 1.42) <0.001		1.21 (1.09, 1.35) <0.001	
Q4 (7.6–17.1)	1,175/1,674	1.31 (1.18, 1.46) <0.001		1.24 (1.12, 1.39) <0.001	
HDL-C status*^f^*					
Q1 (0.6–5.3)	477/2,110	1	0.002	1	0.261
Q2 (5.4–6.3)	529/2,101	1.11 (0.97, 1.28) 0.132		1.04 (0.90, 1.20) 0.588	
Q3 (6.4–7.5)	587/2,213	1.18 (1.03, 1.35) 0.019		1.05 (0.92, 1.21) 0.457	
Q4 (7.6–17.1)	616/2,233	1.23 (1.07, 1.40) 0.003		1.08 (0.94, 1.24) 0.260	

a Model 1: adjusted for sex and age.

b Model 2: adjusted for sex, age, smoking status, alcohol consumption, SBP, DBP, BMI, and diabetes.

c High: TC ≥200 mg/dl, Low: TC <200 mg/dl.

d High: TG ≥150 mg/dl, Low: TG <150 mg/dl.

e High: LDL-C ≥130 mg/dl, Low: LDL-C <130 mg/dl.

f High: HDL-C ≥40 mg/dl, Low: HDL-C <40 mg/dl.

**TABLE 3.** Adjusted ORs (95% CI) for the association between quartiles of lymphocyte count and dyslipidemia by multivariate logistic regression models

Lymphocyte Count (×10^9^ cells/l)	Serum Lipids [Low/High (N)]	Model I*^a^*[OR (95% CI) *P*]	*P* for Trend	Model II*^b^*[OR (95% CI) *P*]	*P* for Trend
TC status*^c^*					
Q1 (0.3–1.5)	825/1,323	1	<0.001	1	<0.001
Q2 (1.6–1.9)	1,098/1,857	1.05 (0.93, 1.18) 0.424		1.03 (0.92, 1.16) 0.611	
Q3 (2.0–2.3)	958/1,685	1.07 (0.95, 1.21) 0.239		1.04 (0.92, 1.17) 0.520	
Q4 (2.4–6.3)	991/2,129	1.29 (1.15, 1.45) <0.001		1.24 (1.10, 1.40) <0.001	
TG status*^d^*					
Q1 (0.3–1.5)	1,543/605	1	<0.001	1	<0.001
Q2 (1.6–1.9)	1,995/960	1.21(1.07, 1.36) 0.003		1.15 (1.01, 1.30) 0.034	
Q3 (2.0–2.3)	1,583/1,060	1.64 (1.45, 1.86) <0.001		1.50 (1.32, 1.71) <0.001	
Q4 (2.4–6.3)	1,670/1,450	2.11 (1.87, 2.38) <0.001		1.82 (1.61, 2.06) <0.001	
LDL-C status*^e^*					
Q1 (0.3–1.5)	973/1,175	1	0.001	1	0.020
Q2 (1.6–1.9)	1,348/1,609	0.99 (0.88, 1.10) 0.819		0.97 (0.87, 1.09) 0.593	
Q3 (2.0–2.3)	1,168/1,475	1.03 (0.92, 1.16) 0.573		1.00 (0.89, 1.13) 0.948	
Q4 (2.4–6.3)	1,273/1,858	1.18 (1.05, 1.32) 0.004		1.12 (1.00, 1.26) 0.047	
HDL-C status*^f^*					
Q1 (0.3–1.5)	356/1,792	1	<0.001	1	<0.001
Q2 (1.6–1.9)	560/2,395	1.18 (1.02, 1.37) 0.026		1.13 (0.97, 1.32) 0.107	
Q3 (2.0–2.3)	579/2,064	1.43 (1.23, 1.65) <0.001		1.32 (1.13, 1.53) <0.001	
Q4 (2.4–6.3)	714/2,406	1.52 (1.32, 1.76) <0.001		1.31 (1.13, 1.52) <0.001	

a Model 1: adjusted for sex and age.

b Model 2: adjusted for sex, age, smoking status, alcohol consumption, SBP, DBP, BMI, and diabetes.

c High: TC ≥200 mg/dl, Low: TC <200 mg/dl.

d High: TG ≥150 mg/dl, Low: TG <150 mg/dl.

e High: LDL-C ≥130 mg/dl, Low: LDL-C <130 mg/dl.

f High: HDL-C ≥40 mg/dl, Low: HDL-C <40 mg/dl.

**TABLE 4.** Adjusted ORs (95% CI) for the association between quartiles of neutrophil count and dyslipidemia by multivariate logistic regression models

Neutrophil Count (×10^9^ cells/l)	Serum Lipids [Low/High (N)]	Model I*^a^*[OR (95% CI) *P*]	*P* for Trend	Model II*^b^*OR (95% CI) P	*P* for Trend
TC status*^c^*					
Q1 (0.3–2.8)	966/1,510	1	<0.001	1	<0.001
Q2 (2.9–3.6)	1,035/1,853	1.16 (1.04, 1.30) 0.009		1.13 (1.01, 1.27) 0.029	
Q3 (3.7–4.5)	921/1,727	1.23 (1.10, 1.38) <0.001		1.18 (1.05, 1.32) 0.006	
Q4 (4.6–13.1)	950/1,904	1.32 (1.18, 1.47) <0.001		1.27 (1.13, 1.42) <0.001	
TG status*^d^*					
Q1 (0.3–2.8)	1,680/796	1	<0.001	1	<0.001
Q2 (2.9–3.6)	1,780/1108	1.36 (1.21, 1.52) <0.001		1.28 (1.14, 1.43) <0.001	
Q3 (3.7–4.5)	1,611/1,037	1.44 (1.28, 1.62) <0.001		1.30 (1.15, 1.46) <0.001	
Q4 (4.6–13.1)	1,720/1,134	1.49 (1.33, 1.67) <0.001		1.38 (1.23, 1.55) <0.001	
LDL-C status*^e^*					
Q1 (0.3–2.8)	1,157/1,319	1	<0.001	1	0.001
Q2 (2.9–3.6)	1271/1,617	1.12 (1.01, 1.25) 0.037		1.09 (0.98, 1.22) 0.104	
Q3 (3.7–4.5)	1,145/1,503	1.17 (1.04, 1.30) 0.006		1.12 (1.00, 1.25) 0.054	
Q4 (4.6–13.1)	1,184/1,670	1.25 (1.12, 1.40) <0.001		1.20 (1.08, 1.34) 0.001	
HDL-C status*^f^*					
Q1 (0.3–2.8)	469/2,007	1	0.106	1	0.628
Q2 (2.9–3.6)	595/2,093	1.11 (0.97, 1.27) 0.136		1.04 (0.91, 1.20) 0.544	
Q3 (3.7–4.5)	547/2,101	1.11 (0.97, 1.27) 0.144		1.01 (0.87, 1.16) 0.912	
Q4 (4.6–13.1)	598/2,256	1.13 (0.99, 1.29) 0.077		1.05 (0.91, 1.21) 0.492	

a Model 1: adjusted for sex and age.

b Model 2: adjusted for sex, age, smoking status, alcohol consumption, SBP, DBP, BMI, and diabetes.

c High: TC ≥200 mg/dl, Low: TC <200 mg/dl.

d High: TG ≥150 mg/dl, Low: TG <150 mg/dl.

e High: LDL-C ≥130 mg/dl, Low: LDL-C <130 mg/dl.

f High: HDL-C ≥40 mg/dl, Low: HDL-C <40 mg/dl.

### Associations of differential leukocyte counts with dyslipidemia profiles by subgroup analysis

We further explored the relationship between total leukocyte, neutrophil, and lymphocyte counts and serum lipids among diabetics. As shown in **Tables 5–7**, subgroup analyses stratified by diabetic status showed that the associations between differential leukocyte counts and high TC and LDL-C remained significant in patients without diabetes (all *P* values <0.001). However, there was no significant modification effect of diabetes on the associations of leukocyte counts with either high TG or low HDL-C risks. Additionally, interaction analyses found significant interaction terms between diabetes and neutrophil counts on TC and LDL-C risks (*P* = 0.033 and *P* = 0.024, respectively).

**TABLE 5.** Adjusted ORs (95% CI) for the association between dyslipidemia and the quartiles of leukocyte count by diabetic status

	Diabetes						
	No			Yes			
Leukocyte Count (×10^9^ cells/l)	Serum Lipids [Low/High (N)]	OR (95% CI) *P*	*P* for Trend	Serum Lipids [Low/High (N)]	OR (95% CI) *P*	*P* for Trend	*P* for Interaction
TC status*^a^*							
Q1 (0.6–5.3)	975/1,348	1	<0.001	71/193	1	0.478	0.552
Q2 (5.4–6.3)	884/1,440	1.17 (1.04, 1.32) 0.009		86/219	0.94 (0.64, 1.37) 0.743		
Q3 (6.4–7.5)	816/1,563	1.35 (1.20, 1.53) <0.001		110/311	1.04 (0.73, 1.50) 0.810		
Q4 (7.6–17.1)	810/1,555	1.36 (1.21, 1.54) <0.001		120/364	1.09 (0.76, 1.55) 0.638		
TG status*^b^*							
Q1 (0.6–5.3)	1,660/663	1	<0.001	159/105	1	<0.001	0.785
Q2 (5.4–6.3)	1,529/795	1.25 (1.10, 1.42) 0.0007		153/152	1.51 (1.07, 2.13) 0.019		
Q3 (6.4–7.5)	1,468/911	1.45 (1.28, 1.65) <0.001		199/222	1.63 (1.18, 2.25) 0.003		
Q4 (7.6–17.1)	1,402/963	1.62 (1.43, 1.84) <0.001		221/263	1.79 (1.31, 2.46) <0.001		
LDL-C status*^c^*							
Q1 (0.6–5.3)	1,150/1,173	1	<0.001	93/171	1	0.497	0.311
Q2 (5.4–6.3)	1,057/1,267	1.16 (1.03, 1.30) 0.014		104/201	1.05 (0.74, 1.50) 0.779		
Q3 (6.4–7.5)	1,023/1,355	1.26 (1.12, 1.41) <0.001		155/266	0.93 (0.67, 1.29) 0.651		
Q4 (7.6–17.1)	1,021/1,344	1.25 (1.11, 1.40) 0.002		154/330	1.15 (0.84, 1.58) 0.405		
HDL-C status*^d^*							
Q1 (0.6–5.3)	424/1,899	1	0.496	53/211	1	0.180	0.519
Q2 (5.4–6.3)	449/1,875	1.00 (0.86, 1.17) 0.951		79/226	1.35 (0.89, 2.03) 0.154		
Q3 (6.4–7.5)	477/1,902	1.02 (0.88, 1.19) 0.801		110/311	1.34 (0.91, 1.96) 0.137		
Q4 (7.6–17.1)	489/1,876	1.05 (0.90, 1.22) 0.517		127/357	1.36 (0.93, 1.98) 0.111		

Adjusted for sex, age, smoking status, alcohol consumption, SBP, DBP, and BMI.

a High: TC ≥200 mg/dl, Low: TC <200 mg/dl.

b High: TG ≥150 mg/dl,Low: TG <150 mg/dl.

c High: LDL-C ≥130 mg/dl, Low: LDL-C <130 mg/dl.

d High: HDL-C ≥40 mg/dl, Low: HDL-C <40 mg/dl.

**TABLE 6.** Adjusted ORs (95% CI) for the association between dyslipidemia and the quartiles of lymphocyte count by diabetic status

	Diabetes						
	No			Yes			
Lymphocyte Count (×10^9^ cells/l)	Serum Lipids [Low/High (N)]	OR (95% CI) *P*	*P* for Trend	Serum Lipids [Low/High (N)]	OR (95% CI) *P*	*P* for Trend	*P* for Interaction
TC status*^a^*							
Q1 (0.3–1.5)	756/1,162	1	0.001	69/161	1	0.119	0.727
Q2 (1.6–1.9)	990/1,582	1.03 (0.91, 1.16) 0.682		108/274	1.09 (0.75, 1.58) 0.657		
Q3 (2.0–2.3)	871/1,422	1.02 (0.89, 1.15) 0.805		87/263	1.25 (0.85, 1.84) 0.254		
Q4 (2.4–6.3)	868/1,740	1.24 (1.09, 1.40) 0.001		123/389	1.29 (0.90, 1.86) 0.159		
TG status*^b^*							
Q1 (0.3–1.5)	1,397/521	1	<0.001	146/84	1	<0.001	0.350
Q2 (1.6–1.9)	1,790/782	1.10 (0.96, 1.26) 0.163		205/177	1.48 (1.05, 2.09) 0.025		
Q3 (2.0–2.3)	1,422/871	1.45 (1.27, 1.67) <0.001		161/189	1.89 (1.33, 2.68) <0.001		
Q4 (2.4–6.3)	1,450/1158	1.80 (1.58, 2.05) <0.001		220/292	2.03 (1.46, 2.83) <0.001		
LDL-C status*^c^*							
Q1 (0.3–1.5)	887/1,031	1	0.020	86/144	1	0.681	0.324
Q2 (1.6–1.9)	1,215/1,357	0.95 (0.84, 1.07) 0.417		132/250	1.13 (0.80, 1.60) 0.496		
Q3 (2.0–2.3)	1,055/1,238	0.98 (0.86, 1.11) 0.732		113/237	1.22 (0.85, 1.74) 0.279		
Q4 (2.4–6.3)	1,094/1,514	1.13 (1.00, 1.28) 0.045		175/337	1.10 (0.78, 1.53) 0.562		
HDL-C status*^d^*							
Q1 (0.3–1.5)	306/1,612	1	<0.001	50/180	1	0.211	0.944
Q2 (1.6–1.9)	470/2,102	1.14 (0.97, 1.34) 0.110		89/293	1.07 (0.71, 1.61) 0.744		
Q3 (2.0–2.3)	489/1,804	1.35 (1.14, 1.59) <0.001		90/260	1.17 (0.78, 1.77) 0.447		
Q4 (2.4–6.3)	574/2,034	1.32 (1.13, 1.55) <0.001		140/372	1.24 (0.84, 1.83) 0.271		

Adjusted for sex, age, smoking status, alcohol consumption, SBP, DBP, and BMI.

a High: TC ≥200 mg/dl, Low: TC <200 mg/dl.

b High: TG ≥150 mg/dl, Low: TG <150 mg/dl.

c High: LDL-C ≥130 mg/dl, Low: LDL-C <130 mg/dl.

d High: HDL-C ≥40 mg/dl, Low: HDL-C <40 mg/dl.

**TABLE 7.** Adjusted ORs (95% CI) for the association between dyslipidemia and the quartiles of neutrophil count by diabetic status

	Diabetes						
	No			Yes			
Neutrophil Count (×10^9^ cells/l)	Serum Lipids [Low/High (N)]	OR (95% CI) *P*	*P* for Trend	Serum Lipids [Low/High (N)]	OR (95% CI) *P*	*P* for Trend	*P* for Interaction
TC status*^a^*							
Q1 (0.3–2.8)	903/1,308	1	<0.001	63/202	1	0.314	0.033
Q2 (2.9–3.6)	948/1,585	1.15 (1.02,1.29) 0.021		87/267	0.93 (0.63,1.37) 0.714		
Q3 (3.7–4.5)	802/1,452	1.25 (1.10, 1.41) <0.001		119/275	0.73 (0.51, 1.05) 0.093		
Q4 (4.6–13.1)	832/1,561	1.32 (1.16, 1.49) <0.001		118/343	0.87 (0.60, 1.25) 0.453		
TG status*^b^*							
Q1 (0.3–2.8)	1,522/689	1	0.001	158/107	1	0.034	0.342
Q2 (2.9–3.6)	1,619/914	1.23 (1.08,1.39) 0.001		161/193	1.67 (1.20,2.33) 0.002		
Q3 (3.7–4.5)	1,424/830	1.26 (1.10, 1.43) <0.001		187/207	1.60 (1.15, 2.21) 0.005		
Q4 (4.6–13.1)	1,494/899	1.36 (1.20, 1.55) <0.001		226/235	1.56 (1.13, 2.14) 0.006		
LDL-C status*^c^*							
Q1 (0.3–2.8)	1,071/1,140	1	<0.001	86/179	1	0.614	0.024
Q2 (2.9–3.6)	1,158/1,357	1.10 (0.98,1.23) 0.107		113/241	1.02 (0.72,1.44) 0.915		
Q3 (3.7–4.5)	987/1,267	1.19 (1.05, 1.34) 0.005		158/236	0.73 (0.52, 1.01) 0.061		
Q4 (4.6–13.1)	1,035/1,358	1.22 (1.09, 1.38) <0.001		149/312	0.99 (0.72, 1.38) 0.972		
HDL-C status*^d^*							
Q1 (0.3–2.8)	419/1,792	1	0.794	50/215	1	0.057	0.167
Q2 (2.9–3.6)	501/2,032	1.00 (0.86,1.16) 0.990		93/261	1.46 (0.97,2.18) 0.067		
Q3 (3.7–4.5)	443/1,811	0.96 (0.82, 1.12) 0.615		104/290	1.43 (0.96, 2.12) 0.079		
Q4 (4.6–13.1)	476/1,917	0.99 (0.85, 1.16) 0.915		122/339	1.55 (1.05, 2.29) 0.026		

Adjusted for sex, age, smoking status, alcohol consumption, SBP, DBP, and BMI.

a High: TC ≥200 mg/dl, Low: TC <200 mg/dl.

b High: TG ≥150 mg/dl,Low: TG <150 mg/dl.

c High: LDL-C ≥130 mg/dl, Low: LDL-C <130 mg/dl.

d High: HDL-C ≥40 mg/dl, Low: HDL-C <40 mg/dl.

## DISCUSSION

In the present cross-sectional study, differential leukocyte counts were consistently and significantly associated with serum HDL-C, LDL-C, TG, and TC levels and increased the odds of high LDL-C, TG, and TC after adjustment for multiple potential confounding variables. Moreover, our study further demonstrated that the associations of total leukocyte, neutrophil, and lymphocyte counts with dyslipidemia were significantly modified by the status of diabetes.

There is compelling evidence that inflammation participates in both the initiation and perpetuation of the atherosclerotic process (23–25). A large-scale longitudinal survey in African-American and Caucasian men and women provided convincing evidence that elevated WBC count was directly associated with increased incidence of coronary heart disease, ischemic stroke, and mortality from CVD (26). Huang et al. (27) reported that in male nonsmokers, the presence of carotid atherosclerosis was significantly associated with total leukocyte count, although there was no significant association in female nonsmokers. Data from a relatively small sample size (N = 264) of participants with suspected coronary artery disease conducted by Rasouli et al. (28) showed that the frequency and severity of coronary artery disease, as well as the prevalence of diabetes mellitus and smoking, were significantly higher in the third tertile of leukocyte count relative to the first tertile. A dose-response association between smoking, serum glucose, TG, hsCRP, and other risk factors, and leukocyte count was found: the higher the smoking, serum glucose, TG, or hsCRP levels, the greater the leukocyte count. Moreover, leukocyte count interacted on a multiplicative scale with smoking, hypertension, and diabetes to increase the risk of coronary heart disease (28). Similarly, our findings showed (supplemental Tables S1–S3) that cigarette smoking can contribute to increased differential leukocyte counts. However, no interactive effects of differential leukocyte counts by smoking status on dyslipidemia risks were observed (data not shown).

Using data from a cohort of 6,328 individuals, the Framingham Heart Study Cohort and Offspring reported a relationship between lipoprotein cholesterol levels and leukocyte count (29). The authors found that elevated leukocyte count was associated with decreased HDL-C and increased LDL-C and VLDL-C levels. In addition to HDL-C and LDL-C levels, our present study further identified that TC and TG levels were also consistently and positively associated with total leukocyte, neutrophil, and lymphocyte counts, suggesting possible ethnic differences in lipid-specific associations with total leukocyte, neutrophil, and lymphocyte counts. In addition, the age of sampling and current smoking and alcohol intake prevalence rates between the Framingham study and our present study are all pronouncedly different. A relatively large study performed by Oda et al. (30) in 2,953 apparently healthy Japanese patients found that total leukocyte count was significantly associated with high LDL-C, high TG, and low HDL-C levels in men. Our findings, based on the largest survey ever conducted in a community-based population, were consistent with the results from Oda et al. (18). Differential leukocyte counts were significantly associated with serum HDL-C, LDL-C, TG, and TC levels, and with an increased odds of high LDL-C, TG, and TC in a Chinese mild-to-moderate hypertensive population. However, in our present population, we detected no obvious significant trends between NLR and lipid profiles (supplemental Table S9).

Similar to differential leukocyte counts, baseline TC, TG, and LDL-C levels were positively associated with erythrocyte or platelet counts after adjusting for multiple covariables, while baseline HDL-C level was marginally and inversely associated with platelet or erythrocyte counts (shown in supplemental Tables S7, S8). The National Health and Nutrition Examination Survey (NHANES) independently examined the relationship between serum cholesterol and circulating erythrocyte/platelet indices on 4,469 adult participants from 2005 to 2006 and on 5,318 adult participants from 2007 to 2008. Consistent with our findings, serum non-HDL-C, such as TC, was positively associated with mean erythrocyte and platelet count (30). Platelet activation plays a key role in the pathogenesis of atherothrombosis and acute coronary syndrome (31). Previous epidemiological studies found that LDL-C enhances platelet activation, leads to platelet hyperactivity, and subsequently increases the risk of arterial thrombosis (32). However, the HDL-C effect on platelet activation is controversial. Our findings showed only a marginal association between erythrocytes and HDL-C. Previous studies have shown that high erythrocyte count can potentially play an atheroprotective role in patients with coronary atherosclerosis. It has been shown that HDL-C levels are directly related to erythrocyte count and inversely related to the prevalence and extent of coronary disease (33). This is in opposition to our findings where TC, TG, and LDL-C levels were positively associated with erythrocytes, and HDL-C level was marginally and inversely associated with erythrocytes. The underlying mechanism has yet to be elucidated.

Furthermore, our study also demonstrated that elevated leukocyte counts were more significantly associated with increased odds of high TC and LDL-C in patients without diabetes, rather than in patients with diabetes. Based on spline smoothing plots of various lipid profiles by glucose levels, we found that, especially for TC and LDL-C levels, when blood glucose levels were less than 8 mmol/l, TC and LDL-C were linearly correlated to glucose levels. At glucose levels above 8 mmol/l, TC and LDL-C levels reached a plateau and were not associated with differential leukocyte counts (as shown in supplemental Fig. S1). In a cross-sectional survey including 5,260 participants from the National Health and Nutrition Examination Survey, the authors demonstrated that the positive association between WBC count and peripheral artery disease risk was dependent on diabetes and smoking status (34). One plausible explanation is that diabetes most likely imposes a more pronounced and variable effect on the pathogenesis of dyslipidemia, rendering the correlation between higher WBC count and blood lipids difficult to detect. Previous studies (34) and our data commonly showed that differential leukocyte counts were higher in those with diabetes. The higher differential leukocyte counts in these hypertensive patients may mask the relationship with dyslipidemia. However, unlike the NHANES study, our study results did not demonstrate any effect modification of the association between WBC count and dyslipidemia by smoking (data not shown).

A strength of our current study is that, to date, it is the largest study to assess an association of differential leukocyte counts with vascular risk factors. Moreover, our population is ideal in that it is a natural representation with only a 3.93% rate of anti-platelet and lipid lowering drug usage. In the final analyses, all treated patients were omitted to ensure reliability of our findings. It is anticipated that once the interactions between lipids and inflammatory factors are better understood, current anti-inflammatory approaches that address low-grade inflammation will be combined with lipid lowering approaches to provide optimal treatment regimens for atherosclerosis. Of course, several limitations are also present: *1*) As a cross-sectional study, the causes of the correlations between elevated differential leukocytes on the risk of developing dyslipidemia cannot be elucidated. Additional prospective studies on the relationship between elevated differential leukocyte counts and vascular diseases including dyslipidemia is warranted in different populations. *2*) Data on some inflammatory cytokines such as CRP, IL-1β, and monocytes were unavailable. Ruan and colleagues found that these inflammatory cytokines significantly increased native LDL-C accumulation in vascular smooth muscle cells specifically via the LDL receptor pathway (35, 36). We recognize that this would have been an important association to examine in our study, had data been available. *3*) HDL subclass measurements were not available, preventing us from predicting a more in-depth risk assessment of various HDL-C subclasses by inflammatory biomarkers, which would allow us to further provide more valuable information to assist in risk stratification of CVD. Further exploratory studies are needed and we aim to address all of these limitations in an effort to verify our results in our ongoing project (CSPPT-2).

In conclusion, we found that elevated leukocyte counts were significantly associated with continuous lipid profiles including serum TC, TG, LDL-C, and HDL-C levels, and with an increased odds of high LDL-C, TG, and TC in Chinese hypertensive patients. Moreover, the presence of diabetes in hypertensive patients may mask the relationship between differential leukocyte counts and dyslipidemia.

## Supplementary Material

Supplemental Data
