# Supplementary material for: Association of peripheral differential leukocyte counts with dyslipidemia risk in Chinese patients with hypertension: insight from the China Stroke Primary Prevention Trial
Source: J Lipid Res. 2016 Dec 29;58(1):256–66. doi: 10.1194/jlr.P067686 (PMC5234728; doi:10.1194/jlr.P067686)
Supplement: Supplemental Data [file supp_58_1_256__index.html]

Association of Peripheral Differential Leukocyte Counts with Dyslipidemia Risk in Chinese Patients with Hypertension: Insight from the CSPPT — Association of peripheral differential leukocyte counts with dyslipidemia risk in Chinese patients with hypertension: insight from the China Stroke Primary Prevention Trial — Supplemental Data 

# Association of peripheral differential leukocyte counts with dyslipidemia risk in Chinese patients with hypertension: insight from the China Stroke Primary Prevention Trial

## Supplemental Data

- Supplemental Data (.pdf, 825 KB) - Supplemental Tables S1-S9 and Figure S1
