# Supplementary material for: Association of peripheral differential leukocyte counts with dyslipidemia risk in Chinese patients with hypertension: insight from the China Stroke Primary Prevention Trial
Source: J Lipid Res. 2016 Dec 29;58(1):256–66. doi: 10.1194/jlr.P067686 (PMC5234728; doi:10.1194/jlr.P067686)
Supplement: Supplemental Data [file 10.1194_P067686_jlr.P067686-1.pdf]

Supplemental tables and figures

Supplemental Table S1. Demographic and clinical parameters by quartiles of white blood cell count.

Variables	WBC quartiles（$\times10^9$cells/L）				P
	Q1(0.6-5.3, n=2587)	Q2(5.4- 6.3, n=2630)	Q3(6.4- 7.5, n=2800)	Q4(7.6-17.1,n=2849)	
**Anthropometrics:**					
Age (years)	58.7 ±7.4	59.2 ±7.6	59.9 ±7.5	60.1 ±7.7	<0.001
SBP (mmHg)	167.2 ±20.3	167.8 ±20.9	168.4 ±20.8	169.0 ±21.2	0.012
DBP (mmHg)	94.7 ±11.5	95.1 ±11.9	95.3 ±11.7	95.0 ±12.2	0.317
BMI (Kg/m^2)	25.1 ±3.4	25.5 ±3.4	25.8 ±3.6	25.8 ±3.8	<0.001
Lymphocyte (10^9/L)	1.6 ±0.4	1.9 ±0.4	2.2 ±0.5	2.6 ±0.8	<0.001
Neutrophil (10^9/L)	2.5 ±0.6	3.3 ±0.5	4.1 ±0.6	5.7 ±1.8	<0.001
Platelet (10^9/L)	221.2 ±75.5	245.2 ±67.8	262.6 ±80.7	292.7 ±114.1	<0.001
RBC (10^{12}/L)	4.5 ±0.7	4.6 ±0.6	4.7 ±0.6	5.0 ±0.8	<0.001
CREA(μmol/L)	64.4 ±15.5	64.7 ±17.5	65.6 ±18.4	65.5 ±22.7	0.067
GLU (mmol/L)	6.0 ±1.6	6.0 ±1.7	6.1 ±1.7	6.2 ±2.1	<0.001
Albumin (g/L)	49.4 ±5.7	49.3 ±5.6	49.1 ±5.7	49.0 ±5.4	0.085
TC (mg/dL)	213.2 ±43.8	217.5 ±45.8	220.3 ±44.0	222.8 ±46.6	<0.001
TG (mg/dL)	131.0 ±60.9	140.4 ±64.6	147.5 ±65.2	154.3 ±70.8	<0.001
LDL-C (mg/dL)	134.5 ±39.7	138.2 ±41.3	140.0 ±40.1	141.9 ±42.3	<0.001
HDL-C (mg/dL)	52.7 ±14.4	51.4 ±13.8	51.0 ±14.3	50.4 ±13.5	<0.001
Diabetes n, (%)	264 (10.2%)	305 (11.6%)	421 (15.0%)	484 (17.0%)	<0.001
Sex, n (%)					0.003
Male	911 (35.2%)	1023 (38.9%)	1116 (39.9%)	1107 (38.9%)	
Female	1676 (64.8%)	1607 (61.1%)	1684 (60.1%)	1742 (61.1%)	
Smoking status, n (%)					<0.001
Never	1964 (75.9%)	1869 (71.1%)	1931 (69.0%)	1924 (67.5%)	
Former	175 (6.8%)	240 (9.1%)	214 (7.6%)	195 (6.8%)	
Current	448 (17.3%)	520 (19.8%)	654 (23.4%)	730 (25.6%)	
Alcohol consumption, n (%)					0.001
Never	1930 (74.7%)	1881 (71.5%)	1936 (69.1%)	2053 (72.1%)	
Former	142 (5.5%)	170 (6.5%)	210 (7.5%)	193 (6.8%)	
Current	513 (19.8%)	578 (22.0%)	654 (23.4%)	603 (21.2%)	
**Previous medication, n (%):**					
Antihypertensive drugs	1192 (46.1%)	1250 (47.5%)	1345 (48.0%)	1416 (49.7%)	0.062
Beta-blocker	17 (0.7%)	22 (0.8%)	31 (1.1%)	33 (1.2%)	0.189
Diuretics	38 (1.5%)	54 (2.1%)	78 (2.8%)	82 (2.9%)	0.001
Angiotensin II Receptor Blockers	5 (0.2%)	2 (0.1%)	2 (0.1%)	3 (0.1%)	0.516
Calcium Channel Blockers	142 (5.5%)	175 (6.7%)	176 (6.3%)	200 (7.0%)	0.124

ACE-inhibitors	233 (9.0%)	211 (8.0%)	232 (8.3%)	222 (7.8%)	0.404
Glucose-lowering drugs	26 (1.0%)	36 (1.4%)	54 (1.9%)	61 (2.1%)	0.003

Abbreviations: DBP, diastolic blood pressure; SBP, systolic blood pressure; BMI, body mass index; RBC, red blood cell; GLU,glucosamine; TC, total cholesterol; TG, triglycerides; LDL-C, low-density lipoprotein cholesterol; HDL-C, high-density lipoprotein cholesterol; WBC, white blood cell; ACE, angiotensin converting enzyme; CREA, Creatinine.

Supplemental Table S2. Demographic and clinical parameters by quartiles of lymphocyte count.

Variables	Lymphocyte count (×10^9cells/L)				P
	Q1(0.3- 1.5, n=2148)	Q2(1.6- 1.9, n=2955)	Q3(2.0- 2.3, n=2643)	Q4(2.4-6.3, n=3120)	
**Anthropometrics:**					
Age (years)	60.1 ±7.8	59.4 ±7.5	59.1 ±7.6	59.6 ±7.4	<0.001
SBP (mmHg)	169.7 ±21.0	168.2 ±20.7	167.4 ±20.5	167.6 ±21.0	<0.001
DBP (mmHg)	95.8 ±12.0	95.1 ±11.9	95.0 ±11.5	94.4 ±11.9	<0.001
BMI (Kg/m^2)	24.8 ±3.3	25.3 ±3.5	25.8 ±3.6	26.2 ±3.7	<0.001
Neutrophil (10^9/L)	3.6 ±1.5	3.7 ±1.3	3.9 ±1.3	4.3 ±1.5	<0.001
Total leukocyte (10^9/L)	5.4 ±1.6	6.0 ±1.5	6.6 ±1.4	7.9 ±1.9	<0.001
Platelet (10^9/L)	224.4 ±73.0	246.4 ±85.0	259.3 ±90.6	285.4 ±98.0	<0.001
RBC (10^{12}/L)	4.5 ±0.8	4.6 ±0.6	4.7 ±0.6	4.9 ±0.8	<0.001
CREA(μmol/L)	67.7 ±24.0	65.7 ±18.4	64.0 ±16.4	63.5 ±16.7	<0.001
GLU (mmol/L)	5.9 ±1.5	6.0 ±1.7	6.1 ±1.9	6.2 ±2.0	<0.001
Albumin (g/L)	49.5 ±5.8	49.2 ±5.8	49.2 ±5.4	49.0 ±5.4	0.004
TC (mg/dL)	215.5 ±44.8	217.0 ±45.3	218.3 ±45.2	222.5 ±45.2	<0.001
TG (mg/dL)	129.1 ±61.7	135.8 ±61.8	147.2 ±66.6	158.0 ±69.4	<0.001
LDL-C (mg/dL)	136.6 ±40.4	137.6 ±40.6	138.5 ±40.8	141.5 ±41.6	<0.001
HDL-C (mg/dL)	53.3 ±14.7	52.5 ±14.6	50.6 ±13.5	49.6 ±13.3	<0.001
Diabetes n,(%)	230 (10.7%)	382 (12.9%)	350 (13.2%)	512 (16.4%)	<0.001
Sex, n (%)					
Male	977 (45.5%)	1220 (41.3%)	950 (35.9%)	1010 (32.4%)	<0.001
Female	1171 (54.5%)	1735 (58.7%)	1693 (64.1%)	2110 (67.6%)	
Smoking status,n (%)					
Never	1474 (68.6%)	2047 (69.3%)	1879 (71.1%)	2288 (73.3%)	<0.001
Former	195 (9.1%)	248 (8.4%)	183 (6.9%)	198 (6.3%)	
Current	479 (22.3%)	659 (22.3%)	580 (22.0%)	634 (20.3%)	
Alcohol consumption, n (%)					
Never	1503 (70.0%)	2084 (70.6%)	1902 (72.0%)	2311 (74.1%)	0.013
Former	146 (6.8%)	196 (6.6%)	167 (6.3%)	206 (6.6%)	
Current	498 (23.2%)	673 (22.8%)	574 (21.7%)	603 (19.3%)	
**Previous medication, n (%):**					
Antihypertensive drugs	1019 (47.4%)	1379 (46.7%)	1251 (47.3%)	1554 (49.8%)	0.076
Beta-blocker	16 (0.7%)	19 (0.6%)	24 (0.9%)	44 (1.4%)	0.012
Diuretics	38 (1.8%)	54 (1.8%)	63 (2.4%)	97 (3.1%)	0.002
Angiotensin II Receptor Blockers	2 (0.1%)	2 (0.1%)	4 (0.2%)	4 (0.1%)	0.792
Calcium Channel Blockers	130 (6.1%)	182 (6.2%)	153 (5.8%)	228 (7.3%)	0.083
ACE-inhibitors	192 (8.9%)	262 (8.9%)	202 (7.6%)	242 (7.8%)	0.164

Glucose-lowering drugs	23 (1.1%)	45 (1.5%)	44 (1.7%)	65 (2.1%)	0.038

Abbreviations: DBP, diastolic blood pressure; SBP, systolic blood pressure; BMI, body mass index; RBC, red blood cell; GLU,glucosamine; TC, total cholesterol; TG, triglycerides; LDL-C, low-density lipoprotein cholesterol; HDL-C, high-density lipoprotein cholesterol; WBC, white blood cell; ACE, angiotensin converting enzyme; CREA, Creatinine.

Supplemental Table S3. Demographic and clinical parameters by quartiles of neutrophil count.

Variables	Neutrophil count ($\times 10^9$ cells/L)				P
	Q1(0.3- 2.8, n=2476)	Q2(2.9- 3.6, n=2888)	Q3(3.7- 4.5, n=2648)	Q4(4.6-67.0, n=2854)	
Anthropometrics:					
Age (years)	58.6 ±7.3	59.4 ±7.5	59.8 ±7.5	60.2 ±7.8	<0.001
SBP (mmHg)	166.5 ±20.3	167.7 ±20.5	168.6 ±21.1	169.6 ±21.3	<0.001
DBP (mmHg)	94.3 ±11.4	94.8 ±11.8	95.5 ±11.9	95.4 ±12.1	<0.001
BMI (Kg/m^2)	25.3 ±3.4	25.7 ±3.5	25.8 ±3.6	25.5 ±3.7	<0.001
Total leukocyte (10^9/L)	4.6 ±0.8	5.8 ±0.7	6.8 ±0.8	8.7 ±1.6	<0.001
Lymphocyte (10^9/L)	1.9 ±0.6	2.1 ±0.6	2.1 ±0.6	2.2 ±0.7	<0.001
Platelet (10^9/L)	227.5 ±76.9	248.1 ±70.4	260.0 ±83.4	287.1 ±114.4	<0.001
RBC (10^{12}/L)	4.5 ±0.7	4.7 ±0.6	4.8 ±0.6	4.9 ±0.8	<0.001
CREA(μmol/L)	63.4 ±15.0	64.3 ±16.0	66.3 ±20.4	66.1 ±22.4	<0.001
GLU (mmol/L)	6.0 ±1.6	6.0 ±1.7	6.1 ±1.8	6.1 ±2.0	0.006
Albumin (g/L)	49.3 ±5.6	49.2 ±5.7	49.1 ±5.6	49.2 ±5.5	0.687
TC (mg/dL)	214.4 ±43.8	218.6 ±45.8	219.3 ±44.3	221.7 ±46.4	<0.001
TG (mg/dL)	134.8 ±62.1	143.6 ±66.3	146.3 ±65.4	148.9 ±69.2	<0.001
LDL-C (mg/dL)	135.3 ±40.1	138.8 ±41.2	139.4 ±40.4	141.1 ±41.7	<0.001
HDL-C (mg/dL)	52.3 ±14.2	51.3 ±14.1	50.9 ±14.0	51.0 ±13.9	0.001
Diabetes n, (%)	265 (10.7%)	354 (12.3%)	394 (14.9%)	461 (16.2%)	<0.001
Sex, n (%)					<0.001
Male	783 (31.6%)	1068 (37.0%)	1097 (41.4%)	1209 (42.4%)	
Female	1693 (68.4%)	1820 (63.0%)	1551 (58.6%)	1645 (57.6%)	
Smoking status, n (%)					<0.001
Never	1915 (77.3%)	2091 (72.4%)	1806 (68.2%)	1876 (65.7%)	
Former	165 (6.7%)	233 (8.1%)	215 (8.1%)	211 (7.4%)	
Current	396 (16.0%)	563 (19.5%)	626 (23.6%)	767 (26.9%)	
Alcohol consumption, n (%)					<0.001
Never	1869 (75.5%)	2081 (72.1%)	1841 (69.5%)	2009 (70.4%)	
Former	135 (5.5%)	179 (6.2%)	189 (7.1%)	212 (7.4%)	
Current	471 (19.0%)	626 (21.7%)	618 (23.3%)	633 (22.2%)	
**Previous medication, n (%):**					
Antihypertensive drugs	1150 (46.4%)	1369 (47.4%)	1278 (48.3%)	1406 (49.3%)	0.199
Beta-blocker	20 (0.8%)	30 (1.0%)	18 (0.7%)	35 (1.2%)	0.161
Diuretics	41 (1.7%)	65 (2.3%)	68 (2.6%)	78 (2.7%)	0.051
Angiotensin II Receptor	5 (0.2%)	4 (0.1%)	0 (0.0%)	3 (0.1%)	0.17
Calcium Channel Blockers	141 (5.7%)	199 (6.9%)	152 (5.7%)	201 (7.0%)	0.068
ACE-inhibitors	212 (8.6%)	224 (7.8%)	225 (8.5%)	237 (8.3%)	0.690
Glucose-lowering drugs	20 (0.8%)	45 (1.6%)	54 (2.0%)	58 (2.0%)	0.001

Abbreviations: DBP, diastolic blood pressure; SBP, systolic blood pressure; BMI, body mass index; RBC, red blood cell; GLU,glucosamine; TC, total cholesterol; TG, triglycerides; LDL-C, low-density lipoprotein cholesterol; HDL-C, high-density lipoprotein cholesterol; WBC, white blood cell; ACE, angiotensin converting enzyme; CREA, Creati

Supplemental Table S4. Association between leukocyte count and baseline lipid profiles using multivariate regression.

Leukocytes (10^9/L)	Mean±SD (mg/dL)	Model I	P for trend	Model II	P for trend
		β(SE)P		*β*(SE)P	
	**TC**				
Q1(0.6-5.3)	213.2 ±43.9	0		0	
Q2(5.4- 6.3)	217.5 ±45.8	4.5 (2.0, 6.9) <0.001		3.9 (1.5, 6.3) 0.001	<0.001
Q3(6.7- 7.5)	220.3 ±44.0	7.1 (4.7, 9.5) <0.001	<0.001	5.8 (3.4, 8.1) <0.001	
Q4(7.6-17.1)	222.8 ±46.6	9.5 (7.1, 11.9) <0.001		8.0 (5.6, 10.4) <0.001	
	**TG**				
Q1(0.6-5.3)	131.0 ±60.9	0		0	
Q2(5.4- 6.3)	140.4 ±64.6	10.2(6.7, 13.7) <0.001	<0.001	7.3 (3.9, 10.6) <0.001	<0.001
Q3(6.7- 7.5)	147.5 ±65.2	17.7(14.2,21.2)<0.001		12.5 (9.1, 15.8) <0.001	
Q4(7.6-17.1)	154.3 ±70.8	24.3(20.8,27.8)<0.001		18.8 (15.5, 22.2) <0.001	
	**LDL-C**				
Q1(0.6-5.3)	134.5 ±39.7	0		0	
Q2(5.4- 6.3)	138.2 ±41.3	3.7 (1.5, 5.9) 0.001	<0.001	3.2 (1.0, 5.4) 0.004	<0.001
Q3(6.7- 7.5)	140.0 ±40.1	5.4 (3.2, 7.6) <0.001		4.2 (2.0, 6.3) <0.001	
Q4(7.6-17.1)	141.9 ±42.3	7.1 (5.0, 9.3) <0.001		5.7 (3.5, 7.8) <0.001	
	**HDL-C**				
Q1(0.6-5.3)	52.7 ±14.4	0		0	
Q2(5.4- 6.3)	51.4 ±13.8	-1.3 (-2.1, -0.6) 0.001	<0.001	-0.8 (-1.5, 0.0) 0.040	<0.001
Q3(6.7- 7.5)	51.0 ±14.3	-1.8 (-2.5, -1.0) <0.001		-0.9 (-1.6, -0.2) 0.015	
Q4(7.6-17.1)	50.4 ±13.5	-2.4 (-3.2, -1.7) <0.001		-1.4 (-2.1, -0.7) <0.001	

Model Ⅰ: adjusted for sex and age.

Model Ⅱ: adjusted for sex, age, smoking, alcohol consumption, SBP, DBP, BMI, and diabetes.

Supplemental Table S5. Association between neutrophil count and baseline lipid profiles using multivariate regression.

Neutrophils (10^9/L)	Mean ±SD (mg/dL)	Model I β(SE)P	P for trend	Model II β(SE)P	P for trend
	**TC**				
Q1(0.3-2.8)	214.4 ±43.8	0	<0.001	0	<0.001
Q2(2.9-3.6)	218.6 ±45.8	4.5 (2.1, 6.9) <0.001		3.9 (1.5, 6.2) 0.001	
Q3(3.7-4.5)	219.3 ±44.3	5.5(3.0, 7.9) <0.001		4.2 (1.8, 6.6) 0.001	
Q4(4.6-13.1)	221.7 ±46.4	7.8 (5.4, 10.2) <0.001		6.5 (4.1, 8.9) <0.001	
	**TG**				
Q1(0.3-2.8)	134.8 ±62.1	0	<0.001	0	<0.001
Q2(2.9-3.6)	143.6 ±66.3	10.0 (6.5, 13.5) <0.001		7.3 (3.9, 10.7) <0.001	
Q3(3.7-4.5)	146.3 ±65.4	13.5 (9.9, 17.1) <0.001		9.2 (5.7, 12.6) <0.001	
Q4(4.6-13.1)	148.9 ±69.2	16.4 (12.8, 19.9) <0.001		13.0 (9.6, 16.4) <0.001	
	**LDL-C**				
Q1(0.3-2.8)	135.3 ±40.1	0	<0.001	0	<0.001
Q2(2.9-3.6)	138.8 ±41.2	3.5 (1.3, 5.7) 0.002		3.0 (0.8, 5.1) 0.007	
Q3(3.7-4.5)	139.4 ±40.4	4.3 (2.1, 6.6) <0.001		3.2 (1.0, 5.4) 0.005	
Q4(4.6-13.1)	141.1 ±41.7	6.0 (3.8, 8.2) <0.001		4.7 (2.5, 6.9) <0.001	
	**HDL-C**				
Q1(0.3-2.8)	52.3 ±14.2	0	<0.001	0	0.024
Q2(2.9-3.6)	51.3 ±14.1	-1.0 (-1.8, -0.3) 0.007		-0.5 (-1.3, 0.2) 0.134	
Q3(3.7-4.5)	50.9 ±14.0	-1.5 (-2.3, -0.8) <0.001		-0.8 (-1.5, -0.1) 0.035	
Q4(4.6-13.1)	51.0 ±13.9	-1.4 (-2.2, -0.6) <0.001		-0.8 (-1.5, -0.1) 0.026	

Model Ⅰ: adjusted for sex and age.

Model Ⅱ: adjusted for sex, age, smoking, alcohol consumption, SBP, DBP, BMI, and diabetes.

Supplemental Table S6. Association between lymphocyte count and baseline lipid profiles using multivariate regression.

Lymphocytes (10^9/L)	Mean±SD (mg/dL)	Model I	P for trend	Model II	P for trend
		β(SE)P		β(SE)P	
	**TC**				
Q1(0.3- 1.5)	215.5 ±44.8	0	<0.001	0	<0.001
Q2(1.6- 1.9)	217.0 ±45.3	1.4 (-1.0, 3.9) 0.264		1.0 (-1.4, 3.5) 0.414	
Q3(2.0- 2.3)	218.3 ±45.2	2.3 (-0.2, 4.9) 0.073		1.7 (-0.8, 4.2) 0.193	
Q4(2.4-6.3)	222.5 ±45.2	6.0 (3.6, 8.5) <0.001		4.9 (2.4, 7.4) <0.001	
	**TG**				
Q1(0.3- 1.5)	129.1 ±61.7	0	<0.001	0	<0.001
Q2(1.6- 1.9)	135.8 ±61.8	6.0 (2.4, 9.6) 0.001		4.0 (0.5, 7.4) 0.024	
Q3(2.0- 2.3)	147.2 ±66.6	16.6 (12.9, 20.3) <0.001		12.5 (8.9, 16.0) <0.001	
Q4(2.4- 6.3)	158.0 ±69.4	26.8 (23.2, 30.4) <0.001		20.0 (16.6, 23.6) <0.001	
	**LDL-C**				
Q1(0.3- 1.5)	136.6 ±40.4	0	<0.001	0	0.003
Q2(1.6- 1.9)	137.6 ±40.6	1.0 (-1.2, 3.3) 0.376		0.7 (-1.6, 2.9) 0.557	
Q3(2.0- 2.3)	138.5 ±40.8	1.7 (-0.6, 4.0) 0.155		1.1 (-1.2, 3.4) 0.346	
Q4(2.4- 6.3)	141.5 ±41.6	4.3 (2.1, 6.6) <0.001		3.3 (1.0, 5.5) 0.005	
	**HDL-C**				
Q1(0.3- 1.5)	53.3 ±14.7	0	<0.001	0	<0.001
Q2(1.6- 1.9)	52.5 ±14.6	-0.8 (-1.6, 0.0) 0.046		-0.4 (-1.2, 0.3) 0.243	
Q3(2.0- 2.3)	50.6 ±13.5	-2.6 (-3.4, -1.8) <0.001		-1.9 (-2.7, -1.1) <0.001	
Q4(2.4- 6.3)	49.6 ±13.3	-3.6 (-4.4, -2.9) <0.001		-2.3 (-3.0, -1.6) <0.001	

Model Ⅰ: adjusted for sex and age.

Model Ⅱ: adjusted for sex, age, smoking, alcohol consumption, SBP, DBP, BMI, and diabetes.

Supplemental Table S7. Adjusted odds ratios (95% CI) for the association between quartiles of platelet count and dyslipidemia by multivariate logistic regression models.

Platelet count (10^9/L)	Serum Lipids Low/High (N)	Model I	P for trend	Model II	P for trend
**TC*a**					
Q1(25.0-209.0)	1187/1393	1	<0.001	1	<0.001
Q2(210.0-246.0)	956/1666	1.48 (1.32, 1.65) <0.001		1.45 (1.30, 1.63) <0.001	
Q3(248.0-289.0)	797/1757	1.84 (1.64, 2.06) <0.001		1.83 (1.63, 2.06) <0.001	
Q4(290.0-985.0)	788/1866	1.94 (1.73, 2.17) <0.001		1.91 (1.70, 2.14) <0.001	
**TG*b**					
Q1(25.0-209.0)	1745/835	1	<0.001	1	<0.001
Q2(210.0-246.0)	1664/958	1.17 (1.05, 1.32) 0.007		1.16 (1.03, 1.30) 0.017	
Q3(248.0-289.0)	1584/970	1.22 (1.08, 1.37) <0.001		1.21 (1.07, 1.36) 0.002	
Q4(290.0-985.0)	1563/1091	1.35 (1.20, 1.51) <0.001		1.33 (1.18, 1.50) <0.001	
**LDL-C*c**					
Q1(25.0-209.0)	1346/1234	1	<0.001	1	<0.001
Q2(210.0-246.0)	1159/1463	1.38 (1.23, 1.54) <0.001		1.36 (1.22, 1.52) <0.001	
Q3(248.0-289.0)	1022/1532	1.62 (1.45, 1.81) <0.001		1.61 (1.44, 1.80) <0.001	
Q4(290.0-985.0)	1029/1625	1.68 (1.50, 1.87) <0.001		1.66 (1.48, 1.86) <0.001	
**HDL-C*d**					
Q1(25.0-209.0)	565/2015	1	0.057	1	0.041
Q2(210.0-246.0)	563/2059	0.98 (0.86, 1.11) 0.728		0.98 (0.86, 1.12) 0.776	
Q3(248.0-289.0)	502/2052	0.88 (0.77, 1.01) 0.063		0.87 (0.76, 1.00) 0.056	
Q4(290.0-985.0)	529/2125	0.90 (0.79, 1.03) 0.133		0.89 (0.77, 1.02) 0.106	

Model I: adjusted for sex and age.

Model II: adjusted for sex, age, smoking status, alcohol consumption, SBP, DBP, BMI, and diabetes.

[a]High: TC ≥ 200 mg/dL, Low: TC < 200 mg/dL.

[b]High: TG ≥ 150 mg/dL,Low: TG < 150 mg/dL.

[c]High: LDL-C ≥ 130mg/dL, Low: LDL-C <130mg/dL.

[d]High: HDL-C ≥40 mg/dL, Low: HDL-C < 40 mg/dL.

Supplemental Table S8. Adjusted odds ratios (95% CI) for the association between quartiles of red blood cell count and dyslipidemia by multivariate logistic regression models.

Red blood cell count (10^9/L)	Serum Lipids Low/High (N)	Model I	P for trend	Model II	P for trend
**TC*a**					
Q1(0.8-4.3)	1052/1597	1	<0.001	1	<0.001
Q2(4.3-4.6)	1003/1711	1.19 (1.07, 1.33) 0.002		1.16 (1.04, 1.30) 0.010	
Q3(4.7-5.0)	919/1817	1.52 (1.35, 1.70) <0.001		1.45 (1.29, 1.62) <0.001	
Q4(5.0-9.8)	898/1866	1.73 (1.54, 1.95) <0.001		1.66 (1.47, 1.88) <0.001	
**TG*b**					
Q1(0.8-4.3)	1731/918	1	<0.001	1	<0.001
Q2(4.3-4.6)	1722/992	1.14 (1.02, 1.28) 0.022		1.08 (0.96, 1.21) 0.224	
Q3(4.7-5.0)	1697/1039	1.33 (1.18, 1.49) <0.001		1.20 (1.07, 1.36) 0.002	
Q4(5.0-9.8)	1639/1125	1.62 (1.44, 1.82) <0.001		1.41 (1.24, 1.59) <0.001	
**LDL-C*c**					
Q1(0.8-4.3)	1266/1383	1	<0.001	1	<0.001
Q2(4.3-4.6)	1261/1453	1.11 (1.00, 1.24) 0.055		1.08 (0.97, 1.21) 0.154	
Q3(4.7-5.0)	1136/1600	1.47 (1.32, 1.65) <0.001		1.41 (1.26, 1.58) <0.001	
Q4(5.0-9.8)	1093/1671	1.72 (1.53, 1.93) <0.001		1.64 (1.45, 1.84) <0.001	
**HDL-C*d**					
Q1(0.8-4.3)	542/2108	1	0.794	1	0.024
Q2(4.3-4.6)	532/2182	0.93 (0.82, 1.07) 0.310		0.89 (0.77, 1.02) 0.097	
Q3(4.7-5.0)	550/2186	0.94 (0.82, 1.07) 0.349		0.88 (0.76, 1.01) 0.065	
Q4(5.0-9.8)	586/2178	0.98 (0.85, 1.12) 0.754		0.84 (0.72, 0.97) 0.020	

Model I: adjusted for sex and age.

Model II: adjusted for sex, age, smoking status, alcohol consumption, SBP, DBP, BMI, and diabetes.

[a]High: TC ≥ 200 mg/dL, Low: TC < 200 mg/dL.

[b]High: TG ≥ 150 mg/dL,Low: TG < 150 mg/dL.

[c]High: LDL-C ≥ 130mg/dL, Low: LDL-C <130mg/dL.

[d]High: HDL-C ≥40 mg/dL, Low: HDL-C < 40 mg/dL.

Supplemental Table S9. Adjusted odds ratios (95% CI) for the association between quartiles of NLR and dyslipidemia by multivariate logistic regression models.

NLR	Serum Lipids Low/High (N)	Model I	P for trend	Model II	P for trend
**TC*a**					
Q1(0.3-1.4)	934/1771	1	0.451	1	0.507
Q2(1.4-1.8)	999/1727	0.93 (0.83, 1.04) 0.183		0.91 (0.81, 1.02) 0.105	
Q3(1.8-2.4)	988/1720	0.95 (0.85, 1.06) 0.353		0.94 (0.84, 1.05) 0.286	
Q4(2.4-10.7)	951/1776	1.04 (0.93, 1.16) 0.514		1.03 (0.92, 1.16) 0.603	
**TG*b**					
Q1(0.3-1.4)	1624/1081	1	<0.001	1	<0.001
Q2(1.4-1.8)	1620/1106	1.06 (0.95, 1.18) 0.336		1.04 (0.93, 1.17) 0.485	
Q3(1.8-2.4)	1718/990	0.91 (0.82, 1.02) 0.100		0.90 (0.80, 1.01) 0.077	
Q4(2.4-10.7)	1829/898	0.80 (0.72, 0.90) <0.001		0.84 (0.74, 0.94) 0.002	
**LDL-C*c**					
Q1(0.3-1.4)	1180/1525	1	0.183	1	0.204
Q2(1.4-1.8)	1228/1498	0.95 (0.86, 1.06) 0.387		0.94 (0.85, 1.05) 0.281	
Q3(1.8-2.4)	1171/1537	1.04 (0.93, 1.16) 0.511		1.03 (0.92, 1.15) 0.598	
Q4(2.4-10.7)	1178/1549	1.05 (0.94, 1.17) 0.370		1.05 (0.94, 1.17) 0.418	
**HDL*d**					
Q1(0.3-1.4)	562/2143	1	0.005	1	0.035
Q2(1.4-1.8)	580/2146	1.02 (0.90, 1.17) 0.704		1.01 (0.89, 1.16) 0.831	
Q3(1.8-2.4)	580/2128	1.03 (0.90, 1.17) 0.711		1.02 (0.89, 1.17) 0.779	
Q4(2.4-10.7)	487/2240	0.81 (0.71, 0.93) 0.003		0.85 (0.73, 0.97) 0.021	

NLR: neutrophil count/ lymphocyte count.

Model I: adjusted for sex and age.

Model II: adjusted for sex, age, smoking status, alcohol consumption, SBP, DBP, BMI, and diabetes.

[a]High: TC ≥ 200 mg/dL, Low: TC < 200 mg/dL.

[b]High: TG ≥ 150 mg/dL,Low: TG < 150 mg/dL.

[c]High: LDL-C ≥ 130mg/dL, Low: LDL-C <130mg/dL.

[d]High: HDL-C ≥40 mg/dL, Low: HDL-C < 40 mg/dL.

Supplemental Figure S1. Multivariate smoothing spline plots of baseline lipid profiles by blood glucose levels.

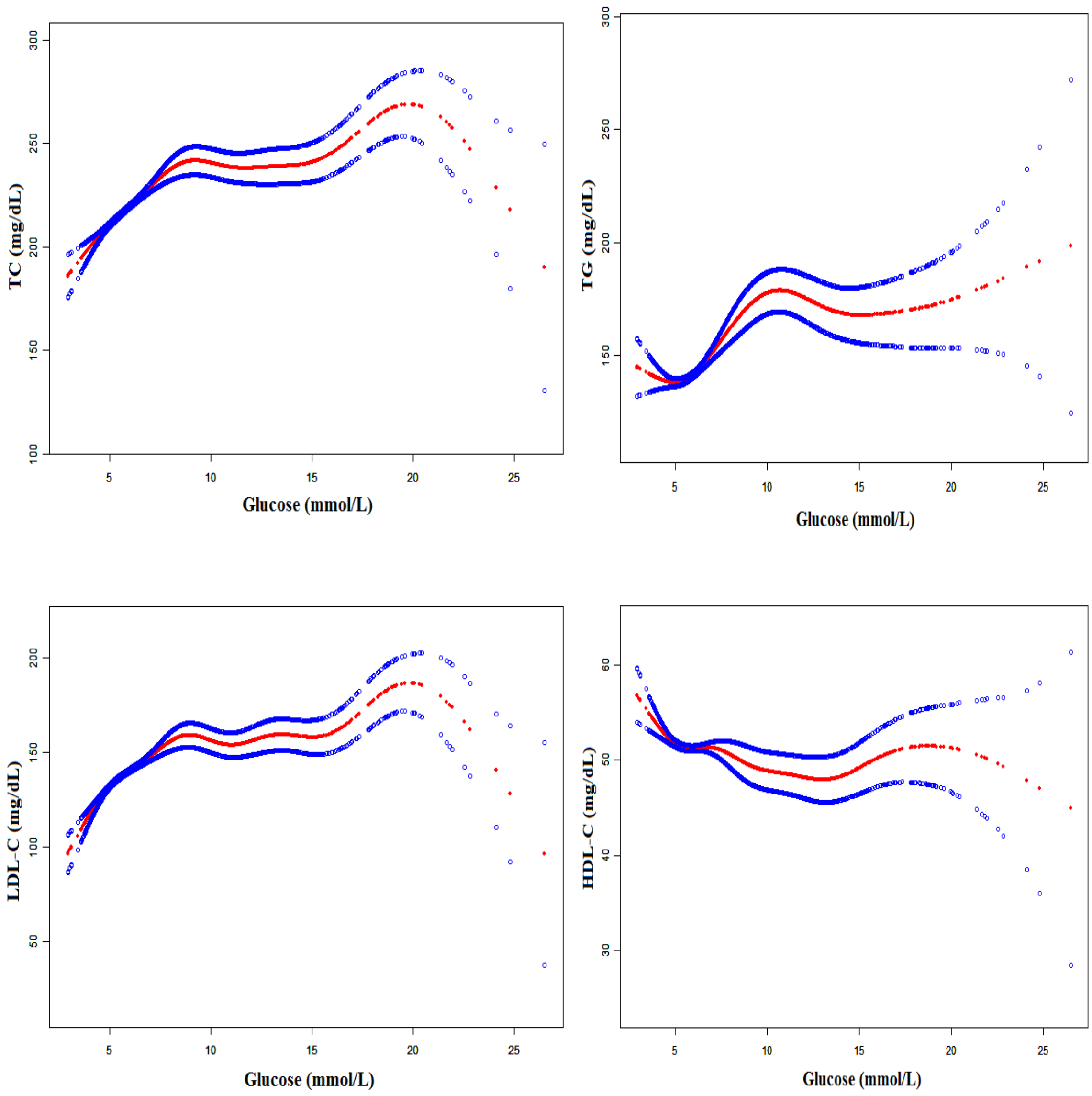

Red dotted lines represent the spline plots of glucose levels and blue dotted lines represent the 95% confidence intervals of the spline plots.

Adjusted for sex, age, smoking status, alcohol consumption, SBP, DBP, and BMI.
